# Attitude of Romanian Medical Students and Doctors toward Business Ethics: Analyzing the Influence of Sex, Age, and Ethics Education

**DOI:** 10.3390/ejihpe13080106

**Published:** 2023-08-09

**Authors:** George-Dumitru Constantin, Crisanta-Alina Mazilescu, Teodora Hoinoiu, Bogdan Hoinoiu, Ruxandra Elena Luca, Loredana-Ileana Viscu, Ioana Giorgiana Pasca, Roxana Oancea

**Affiliations:** 1 Faculty of Dental Medicine, Victor Babes University of Medicine and Pharmacy, 300041 Timisoara, Romania; george.constantin@umft.ro (G.-D.C.); hoinoiu@umft.ro (B.H.); luca.ruxandra@umft.ro (R.E.L.); pasca.ioana@umft.ro (I.G.P.); roancea@umft.ro (R.O.); 2 Teacher Training Department, Politehnica University Timisoara, 300596 Timisoara, Romania; 3 Faculty of Medicine, Victor Babes University of Medicine and Pharmacy, 300041 Timisoara, Romania; tstoichitoiu@umft.ro; 4 Faculty of Psychology, Tibiscus University of Timisoara, 300559 Timisoara, Romania

**Keywords:** attitude, business ethics, ethics education, medical students, doctors

## Abstract

This study investigated the attitude of Romanian medical students and doctors toward business ethics by measuring the preference for a particular ethical philosophy, namely, the preference for Machiavellianism, moral objectivism, social Darwinism, ethical relativism, and legalism. At the same time, this study aimed to explore the influence of sex, age, and ethics education on the attitude toward business ethics. The data collection was performed using a voluntary self-administered online survey including the Attitudes Toward Business Ethics Questionnaire (ATBEQ) instrument. Our findings show that the values based on which Romanian medical students and doctors make business decisions belong predominantly to the moral objectivism philosophy, which is grounded on rational actions based on a set of objective moral standards.

## 1. Introduction

The medical workforce is facing increasingly greater challenges related to the evolution of diseases, an aging population, communication with increasingly difficult people, rigid organizational hierarchies, and new demands for data confidentiality, digitization, and external factors that involve working in a volatile, complex, uncertain, and ambiguous environment. The ability to deal with these new challenges is closely linked to the skills of medical personnel, their ability to be motivated and determined, their ability to cope with stress, to manage their own emotions effectively, and to manage the organizational reality in which they carry out their activities efficiently. All these challenges require strategic, operational, and interpersonal skills.

A sustainable and committed medical workforce requires an integrated ethical and managerial education in medical training. Many medical faculties have included courses in management, organizational behavior, strategy, accounting, economics, and leadership in their medical degree curricula, as well as ethics courses (in this case, the orientation is predominantly toward medical ethics and deontology). Each discipline in particular and all of them together influence the ethical behavior of future doctors, whether it is their ethical behavior in relation to patients or their ethical behavior in an organizational environment or in a medical business context.

In this study, we were interested in the attitude of medical students toward business ethics and the influence of sex, age, and ethics education on the attitude toward business ethics.

The attitude toward business ethics has been studied from the perspective of multiple disciplines (philosophy, psychology, and economics), but most research has focused either on the ethical behavior and judgments of management students or on the comparative analysis of business students’ and nonbusiness students’ attitudes.

Business is essential for the well-being of a society. Business and business ethics are not only the domain of business specialists but also of many other professional categories, such as private sector entrepreneurs or liberal professionals (lawyers, psychologists, doctors, etc.), in which specialists manage their own business in a specific field and have a specific behavior and attitude toward business ethics.

As independent practitioners, doctors have autonomy in making business decisions, which comes with the responsibility to act ethically and be accountable for the impact of their decisions on patient care, financial transactions, and professional reputation. Upholding business ethics ensures that their autonomy is exercised in a manner that prioritizes patient well-being and ethical conduct.

On the other hand, doctors in private practices often have financial interests tied to their businesses. This dynamic introduces the potential for conflicts of interest, where the pursuit of profit may clash with the best interests of patients. By embracing business ethics, doctors can effectively manage conflicts of interest, ensuring that financial considerations never compromise patient care and that decisions are made solely based on medical necessity.

Ethical business practices contribute to a positive reputation, attracting patients, and fostering the sustainability and growth of private practices. Integrating business ethics ensures patient welfare, professional integrity, and upholds ethical standards, leading to a medically excellent and ethically sound healthcare system.

### 1.1. Business Ethics

Over time, different schools of thought, oriented toward certain ethical philosophies, have proposed ideas and interpretations of what is ethical or unethical, moral or immoral, right or wrong. Contextualized in business, ethics is defined either in terms of right and wrong in terms of business activities and decisions [1], or in terms of good and bad, or correct and incorrect behaviors and practices [2]. 

Bageac et al. [3] adopted the perspective of Carroll and Buchholtz [2] and stated that both terms from morality (right and wrong) and ethics (good and bad) can be used in the study of business behavior because, in the business context, these terms are interchangeable. One of the first attempts to quantify ethical philosophies on a multi-dimensional scale was made by Reidenbach and Robin, who developed the Multidimensional Ethics Scale (MES), based on three dimensions: broad-based moral equity, relativistic, and contractualism [4]. Hansen refined the MES and arrived at five decision-making ethical philosophies: deontology, utilitarianism, egoism, relativism, and justice [5]. 

Miesling and Preble compared five business philosophies [6]. Four of these were adopted from Stevens [7], namely, Machiavellianism, objectivism, social Darwinism, and ethical relativism. They added universalism to their analysis.

The Multidimensional Ethics Scale, along with Forsyth’s Ethics Position Questionnaire (EPQ) and Neumann and Reichel’s Attitudes Toward Business Ethics Questionnaire (ATBEQ), are considered to be three of the most important scales used in the business ethical decision-making research [8]. 

The Attitudes Toward Business Ethics Questionnaire (ATBEQ) was initially developed by Neumann and Reichel [9] and is based on the ethical philosophies presented by Stevens [7] and reiterated by Miesling and Preble [6] in their analysis: Machiavellianism, objectivism, social Darwinism, ethical relativism, and legalism. 

Although the results of research on students’ attitudes toward business ethics from different faculties are inconclusive, the general impression tends to be that business students are more tolerant of questionable business practices than nonbusiness students [10] or that business students cheat more or are less cooperative than students in other academic fields [11,12]. 

The first objective of our research was to identify the attitude toward business ethics of medical students by measuring the preference for one philosophy over another, namely, identifying the preference for Machiavellianism, moral objectivism, social Darwinism, ethical relativism, and legalism. 

Moral objectivism is the belief that there are universal moral truths that apply to all individuals and cultures. In the context of business ethics, moral objectivism can provide a foundation for ethical decision making, as it suggests that there are certain moral principles that should guide behavior, regardless of the specific context. In medicine, moral objectivism can provide a framework for ethical decision making that prioritizes the well-being of patients and adherence to professional standards.

Machiavellianism is an attitude that can have significant implications for business ethics, as well as for doctors and medical students. In the context of business ethics, individuals who exhibit high levels of Machiavellianism tend to be characterized by a number of traits, including a willingness to deceive and manipulate others, a tendency to focus on their own self-interests rather than the interests of others or the organization as a whole, and a tendency to view relationships with others as transactions that can be used to achieve their own ends. In the context of medicine, it can lead to medical professionals prioritizing their own interests over the best interests of their patients. 

Social Darwinism is the belief that society should be organized according to the principles of natural selection, with the strongest individuals or groups succeeding and the weakest failing. In the context of business ethics, social Darwinism can lead to a focus on competition and individual success, rather than collaboration and social responsibility. In medicine, it can lead to a focus on individual success rather than the well-being of patients.

Ethical relativism is the belief that moral truths are relative to the individual or culture in which they exist. In the context of business ethics, ethical relativism can lead to a lack of universal ethical standards, as what is considered ethical can vary depending on the context. In medicine, ethical relativism can lead to a lack of universal ethical standards, which can make it difficult to prioritize patient well-being.

Legalism is the belief that ethical behavior is determined by adherence to laws and regulations. In the context of business ethics, legalism can lead to a focus on legal compliance rather than ethical behavior, as individuals may prioritize avoiding legal consequences over doing what is right. In medicine, legalism can lead to a focus on legal compliance rather than patient well-being, as medical professionals may prioritize avoiding legal consequences over doing what is best for their patients.

The attitudes of Machiavellianism, moral objectivism, social Darwinism, ethical relativism, and legalism can all have significant implications for ethical behavior in the contexts of business and medicine. Understanding these attitudes can help individuals and organizations make more informed and ethical decisions.

### 1.2. Ethics Education and Attitude toward Business Ethics

Education and professional experience have been among the variables considered by many researchers who have studied the factors that can influence ethical attitudes. Thus, Alonso-Almeida, Fernandez de Navarrete, and Rodriguez-Pomeda showed that students with a higher level of education also have a higher ethical consciousness [13]. 

Although some literature does not indicate a significant impact of ethics education on students [14,15,16], more and more studies are presenting evidence of the positive effect of ethics education on moral judgment and ethical consciousness [17].

The research of Hermannsdottir, Stangej, and Kristinsson suggests that there is no difference in attitudes toward business ethics between business students and nonbusiness students, meaning that ethical behavior is not predetermined by the students’ professional orientation but rather by ethics education and studying specific subjects [18]. Giacalone [19] suggests that universities should be involved in developing students’ ethical attitudes through ethics education that helps students acquire the knowledge and skills necessary to make correct judgments and develop a sense of ethics [20,21,22]. 

Business ethics is a discipline that is part of the curriculum of only some faculties. It is often found in the curricula of business, management [23,24,25], or accounting students [26,27]. 

In general, students, regardless of their faculty, receive ethics courses. However, most of these are either general ethics courses or courses in professional ethics that are oriented toward a specific university specialization such as engineering ethics [28] or medical ethics [29].

Although the need for management in the medical field is great, the ethical management education of medical students is not a major objective of universities and, as a result, there are few (if any) courses on business ethics. However, medical students take full advantage of courses in ethics and professional ethics, which contribute to forming and strengthening doctors’ ethical behavior [30], which, in turn, reflects on their ethical attitudes in business. 

Reflective ethical development is contributed to by all university courses to a greater or lesser extent through discussions of ethical controversies that are probably more contextualized in a medical care environment than in a business one but that influence the ethical attitudes and behaviors of medical students.

In this study, we were interested in the relationship between university ethics education and students’ attitudes toward business ethics, considering that differences in perceptions of business ethics between medical students and students from other specialties (engineering and management) are due to both the specificity of instruction and education in general as well as the ethics courses taught by the faculty in particular. 

Based on the above, we formulated the following hypotheses:

**Hypothesis** **1.1.** *Based on a different ethics education, there are significant differences in attitude toward business ethics between medical students and management students.*

**Hypothesis** **1.2.** *Medical students and doctors who benefited from similar ethics educations perceive business ethics similarly. There are not significant differences in attitude toward business ethics between medical students and doctors.*

### 1.3. Sex and Attitude toward Business Ethics

The relationship between sex and business ethics has been extensively studied and argued from either the perspective of different socialization of women and men [31,32,33] or from the perspective of men’s competitiveness or women’s need to maintain harmonious relationships [31,34]. To explain the differences identified, some studies considered different personal values [35] or contextualizes the relationship between sex and ethics by considering that cultural norms determine these differences [36,37]. 

In these studies that highlight differences between men and women in terms of business ethics, it is considered that women are more ethical [34,38] or more concerned with ethics [39]. On the other hand, there are also studies that support the idea that there are no significant differences between men and women in terms of moral development or ethical decisions [40,41,42,43,44]. 

Studies concerning students are no different from those presented above. Thus, we find research that did not identify any sex differences among university students in terms of ethical responses [45]. Another study on healthcare professionals’ clinical practice indicates no differences in moral sensitivity between women and men; in terms of ethical sensitivity, women reported significantly higher ethical sensitivity than men [46]. However, most studies highlight differences between women and men in terms of concern for ethical issues in general [47], with women having a higher business ethics than male students [48,49,50].

Based on some of the results of these studies, we formulated the following hypotheses:

**Hypothesis** **2.1.** *There are significant differences in attitude toward business ethics between men and women, both in the group of medical students and in the group of management students.*

**Hypothesis** **2.2.** *There are significant differences in attitude toward business ethics between male and female doctors.*

### 1.4. Age and Attitude toward Business Ethics

Age is an important variable in understanding ethical attitudes, with the relationship between age and moral judgment being described in Kohlberg’s moral development model, a model accepted and recognized by experts [51]. Yamamura and Stedman believe that age has long been considered a critical factor in ethical literature; their research on Japanese business people highlighted the fact that older people have higher levels of ethics than younger people [52]. Other research complements this opinion, with results showing that young people are more inclined to commit unethical acts than older people [33] or that older people have been identified to have higher moral reasoning [53]. 

In general, it is considered that ethical behaviors increase with age [36,54,55], but there are also studies that consider age as a variable that does not significantly influence ethical behavior [33,53].

**Hypothesis** **3.** *Age has a significant impact on the attitude of medical students toward business ethics.*

## 2. Materials and Methods

### 2.1. Participants

This study involved 53 medical students and 192 doctors who graduated from the same medical university and 108 students from a management faculty of a technical university. Out of the total group of 161 students, 38 were men (23.6%) and 123 were women (76.4%). The average age of the 3 groups studied was 31.5 years for the group of doctors, 24.4 years for medical students, and 20.8 years for management students.

### 2.2. Instrument

The Attitudes Toward Business Ethics Questionnaire (ATBEQ) was used as the measuring instrument to investigate ethical attitudes [56]. Respondents were asked to express their opinions on a range of ethical issues presented in the form of 30 practical scenarios and to rate their agreement level with each question on a five-point Likert scale from 1 (totally disagree) to 5 (totally agree). In addition to the standard application, respondents were asked to analyze the practical scenarios from the perspective of their professional field of activity. The ATBEQ has a reliability of 0.807. 

In previous research studies utilizing the ATBEQ scale, good reliabilities have been reported, such as 0.710 [57], 0.80 [58] and 0.972 [59].

### 2.3. Data Collection and Ethical Consideration

The data collection process for this study involved obtaining information from participants through a structured online questionnaire. Participants were recruited through targeted sampling techniques, such as reaching out to students and healthcare professionals via professional networks, organizations, and educational institutions and through their social media. The data collection period lasted three months, during which participants received two invitations to participate in the study, along with clear instructions and assurances of confidentiality.

Ethical considerations were of utmost importance throughout the study. Informed consent was obtained from all participants, ensuring that they were fully aware of the study’s purpose, procedures, potential risks, and benefits, and that their participation was voluntary. Participant anonymity and confidentiality were strictly maintained throughout the study and any identifying information was kept separate from the collected data. Additionally, participants were given the freedom to withdraw from the study at any point without penalty or consequence. 

### 2.4. Data Analyses

An analysis of variance (ANOVA) was conducted to highlight the differences between the predominant ethical philosophies among groups of medical and management students as well as between medical students and doctors. Additionally, ANOVA was used to explore differences based on sex, ethical education, and age and the five subdimensions of business ethics. 

A repeated measures analysis of variance (ANOVA) was conducted to investigate potential differences among the five dimensions of business ethics for medical students and separately in the group of doctors.

A multiple regression analysis was performed to examine the relationship between ethics education, sex, age, and the five subdimensions of business ethics with a sample comprising both medical and management students. The five subdimensions analyzed were Machiavellianism, Ethical relativism, moral objectivism, social Darwinism, and legalism.

## 3. Results

### 3.1. Business Philosophies Guiding the Behavior of Medical Students

Attitudes toward business ethics were measured through five subdimensions (business philosophies) by measuring the preference for one philosophy or another, namely, the preference for Machiavellianism, moral objectivism, social Darwinism, ethical relativism, and legalism. The hierarchy of results obtained is as follows: moral objectivism (M = 3.18), Machiavellianism (M = 2.52), ethical relativism (M = 2.40), social Darwinism (M = 2.37), and legalism (M = 2.38). Table 1 presents the mean values and standard deviations regarding agreement with the five ethical philosophies for the group of medical students. 

Moral objectivism is a philosophy based on rational actions aimed at achieving one’s own well-being by conforming to a set of objective moral standards, considered valid for all people and situations, regardless of culture, beliefs, or feelings. Achieving one’s own well-being or realizing one’s own interest does not occur at any cost. It is considered that through rational decisions, productivity and happiness can be achieved. Moral objectivism considers that the real world and one’s own interests are not in contradiction to ethics [6]. 

A repeated measures analysis of variance (ANOVA) with one within-subjects factor was conducted to determine whether significant differences existed among the five dimensions analyzed for business ethics for medical students.

The main effect for the within-subjects factor was significant, *F*(4, 208) = 22.88, *p* < 0.001, indicating there were significant differences between the values of Machiavellianism, ethical relativism, moral objectivism, social Darwinism, and Legalism. 

Tukey’s comparison test, with an alpha of 0.05, was used to determine these differences. 

Machiavellianism was significantly less than moral objectivism (MO) (*t*(52) = −8.30, *p* < 0.001); ethical relativism (ER) was significantly less than moral objectivism (MO) (*t*(52) = −7.87, *p* < 0.001); social Darwinism was significantly less than moral objectivism (MO) *t*(52) = −9.64, *p* < 0.001); and moral objectivism (MO) was significantly greater than legalism (*t*(52) = 6.23, *p* < 0.001). 

**Figure 1:**
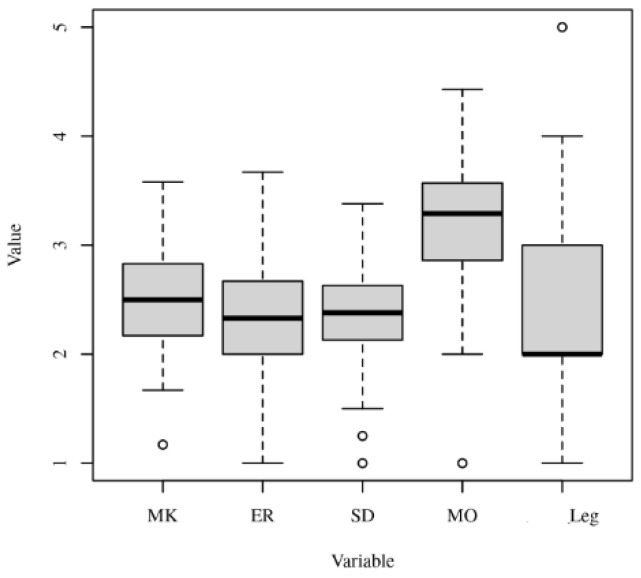
Boxplots of Machiavellianism, ethical relativism, social Darwinism, moral objectivism, and legalism in the medical students group.

The values that medical students rely on when making business decisions were predominantly based on the philosophy of moral objectivism (Figure 1). Although Machiavellianism ranked second, the preference for Machiavellianism was low, and the difference between moral objectivism and Machiavellianism was statistically significant. Practically speaking, we can say that there is a dominant philosophy that underlies ethical behavior in business for medical students and that is the philosophy of moral objectivism.

Next, we analyzed the attitude toward business ethics in the group of doctors. A repeated measures analysis of variance (ANOVA) was conducted to determine whether significant differences existed among the five dimensions analyzed for business ethics in the doctor group. 

Table 2 presents the mean values and standard deviations regarding agreement with the five ethical philosophies for the doctor group. 

**Figure 2:**
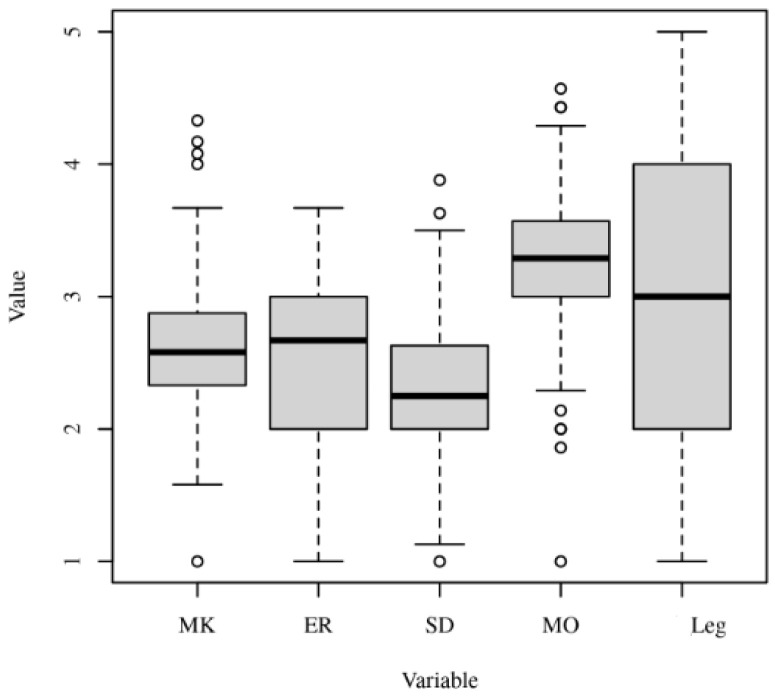
Boxplots of Machiavellianism (MK), ethical relativism (ER), social Darwinism (SD), moral objectivism (MO), and legalism (Leg) for the doctors’ group.

The results indicate that there were significant differences (*F*(4, 764) = 64.53, *p* < 0.001) between the Machiavellianism, ethical relativism, social Darwinism, moral objectivism, and legalism in the doctors’ group. The distribution of the results are visible in the Figure 2.

From the results obtained, we can say that, for doctors, the preferred business philosophy was moral objectivism, followed by legalism, then Machiavellianism and ethical relativism, and lastly social Darwinism.

### 3.2. Ethics Education and Attitude toward Business Ethics

Education is based on well-established values and is oriented by educational objectives, goals, and ideals. Throughout history, philosophers have continuously demonstrated that ethics is intrinsic to any educational action [60].

The school does not prepare exclusively for the profession required by the needs of society [61] but transmits some values that guide behavior.

The groups of students participating in the study were selected from two universities, one university with a medical profile and another with a management and technical profile. The ethics and university deontology and management courses are common courses, included in the curriculum of all students of this university, and the courses were taught by the same team of teachers and had a similar syllabus.

Being interested in the connection between university ethics education and students’ attitude toward business ethics, we considered that the business attitude of medical students differs from that of management students, and these differences are due to the ethics courses taught by the faculty/university.

Taking into account these clarifications, we will continue to consider the ethics education variable as being specific to each university.

We examined whether medical students are different from management students in the five dimensions of business ethics. 

**Figure 3:**
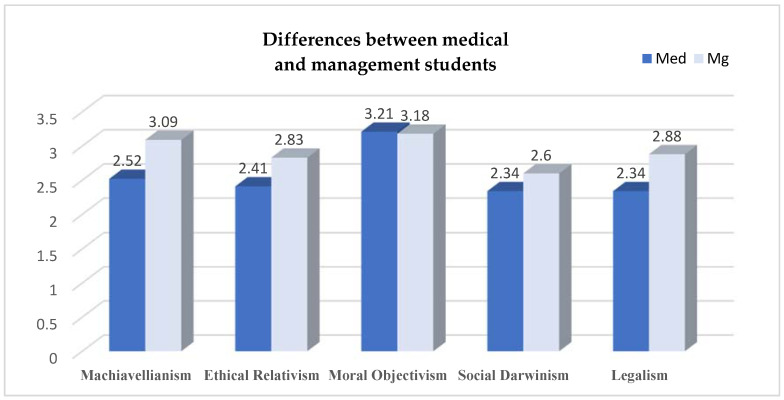
Differences in attitude toward business ethics between medical and management students.

Figure 3 shows the differences regarding the ethical attitude in business between the two groups of students, medical students and management students, differences reflected in the dimensions of Machiavellianism, ethical relativism, moral objectivism, social Darwinism, and legalism.

An ANOVA was performed to examine the variations in business philosophies based on ethics education/faculty among medical and management students (Appendix A Table A1). The ANOVA yielded significant results for four of the five dimensions studied, specifically Machiavellianism, ethical relativism, social Darwinism, and legalism.

- Machiavellianism (MV): *F*(1, 159) = 56.69, *p* < 0.001;
- Ethical relativism (ER): *F*(1, 159) = 19.90, *p* < 0.001;
- Social Darwinism (SD): *F*(1, 159) = 10.39, *p* = 0.002;
- Legalism (Leg): *F*(1, 159) = 9.12, *p* = 0.003.

Concerning moral objectivism, there were no significant differences between medical students and management students (*F*(1, 159) = 0.42, *p* = 0.519).

These findings confirm Hypothesis 1.1, which posited that significant differences exist between medical and management students in their attitudes toward business ethics, except for their attitudes toward moral objectivism.

Next, we were interested in comparing the ethical business attitude of the medical students to that of the group of doctors participating in the study, given that the doctors were graduates of the same medical university as the medical students. We thus took into account that both students and doctors benefited from an ethics education/instruction coming from the same university.

The ANOVA results revealed no significant differences in four of the five dimensions of attitude toward business ethics between the medical students and the doctors. These dimensions were Machiavellianism, ethical relativism, social Darwinism, and moral objectivism. 

However, there was a significant difference in legalism between the two groups, indicating that the medical students had a significantly higher mean legalism score than the doctors (*F*(1, 256) = 4.51, *p* = 0.035). 

These findings confirm Hypothesis 1.2, which posited that there are no significant differences between doctors and medical students in their attitudes toward business ethics, except for their attitudes toward legalism, where significant differences were identified.

### 3.3. Sex and Attitude toward Business Ethics in the Medical and the Management Student Groups

We hypothesized that there are significant differences in attitude toward business ethics between male and female students in both the medical and management groups.

For this purpose, we performed an ANOVA on the group of medical students and separately on the group of management students.

The results indicate that there were no significant differences between male and female medical students for any of the dimensions of business ethics, more precisely for any of the Machiavellianism, ethical relativism, moral objectivism, social Darwinism, and legalism dimensions. 

Regarding male and female management students, the results of ANOVA showed that there were significant differences in Machiavellianism (F(1, 105) = 5.80, *p* = 0.018, eta squared = 0.05), with a larger mean for men (M = 3.23, SD = 0.46) than for women (M = 3.02, SD = 0.39). Sex explained approximately 5% of the variance in Machiavellianism. 

There were also significant differences in social Darwinism (F(1, 105) = 6.22, *p* = 0.014, eta squared = 0.06), with sex explaining approximately 6% of the variance. 

However, the ANOVA results for ethical relativism, moral objectivism, and legalism were not significant, indicating that there were no significant differences in these variables between male and female management students. Therefore, it can be concluded that sex had a significant effect on Machiavellianism and social Darwinism but not on ethical relativism, moral objectivism, and legalism.

Based on the results provided, Hypothesis 2.1, stating that there are significant differences in attitude toward business ethics between male and female students in both the medical and management groups, was partially supported. Specifically, the results indicate that there were no significant differences for any of the dimensions of business ethics between male and female medical students, but there were significant differences in Machiavellianism and social Darwinism between male and female management students, with sex explaining approximately 5% and 6% of the variance, respectively. However, there were no significant differences in ethical relativism, moral objectivism, or legalism between male and female management students.

Therefore, Hypothesis 2.1 can only be partially supported, as it was supported for one group (management students) but not for the other (medical students).

We continued our study by analyzing the differences between male and female doctors in terms of their attitude toward business ethics. 

Based on the results obtained, it appears that there were no significant differences between male and female doctors in terms of their attitudes toward business ethics. This suggests that Hypothesis 2.2, which predicted significant differences between male and female doctors in this area, is not supported by the data.

### 3.4. Age and Attitude toward Business Ethics

The ANOVA results indicate that there were no significant differences in attitude toward business ethics between different age groups of medical students for the measures of moral objectivism, Machiavellianism, ethical relativism, social Darwinism, or legalism.

Based on the results provided, it appears that Hypothesis 3, which stated that age has a significant impact on the attitude of medical students toward business ethics, was not supported by the data.

### 3.5. Multiple Regression Analysis according to Business Ethics

We tested the relationship among ethics education, sex, and age and the five subdimensions of business ethics. The regression analysis was conducted on a sample comprising both medical and management students. 

Machiavellianism (MK): Ethics education and sex significantly predicted Machiavellianism. Ethics education was associated with lower levels of MK, indicating that individuals with greater exposure to ethics education exhibited decreased Machiavellian tendencies (B = −0.57, t(311) = −7.76, *p* < 0.001). Additionally, women scored lower on Machiavellianism than men (B = −0.18, t(311) = −3.18, *p* = 0.002). Age did not have a significant effect on MK.

Social Darwinism (SD): Ethics education and sex significantly predicted social Darwinism (F(3, 311) = 13.12, *p* < 0.001, R2 = 0.11). Greater ethics education was linked to lower levels of social Darwinism(SD) (B = −0.26, t(311) = −4.00, *p* < 0.001). Women showed lower levels of SD than men (*B* = −0.20, t(311) = −4.00, *p* < 0.001). Age did not significantly predict SD.

Moral Objectivism (MO): None of the tested variables (ethics education, sex, and age) significantly predicted moral objectivism.

Ethical Relativism (ER): Ethics education significantly predicted ethical relativism, with greater education associated with lower levels of ER. Sex and age were not significant predictors of ER.

Legalism: Ethics education significantly predicted legalism, with higher education associated with lower levels of legalism (*B* = −0.66, *t*(311) = −3.71, *p* < 0.001). Sex and age did not significantly predict legalism.

Overall, the results of the regression analysis suggest that ethics education and sex are important predictors of several subdimensions of business ethics for medical and management students.

Greater exposure to ethics education was associated with lower levels of machiavellianism, social darwinism, ethical relativism, and legalism. Women consistently demonstrated lower scores on Machiavellianism and social Darwinism. Age did not play a significant role in predicting any of the subdimensions. These findings underscore the significance of integrating ethics education and sex considerations into business ethics training programs.

## 4. Discussion

This study analyzed the attitude of Romanian medical students and doctors toward business ethics, as well as the influence of sex, age, and ethics education. 

The attitudes toward business ethics were measured through five subdimensions, namely, their preference for machiavellianism, moral objectivism, social darwinism, ethical relativism, and legalism. 

The dominant philosophy underlying ethical behavior in business for medical students was found to be moral objectivism. This indicates a preference for rational actions aimed at achieving individual well-being while conforming to universal moral standards. This finding might be attributed to the ethics courses provided in medical curricula, which instill a strong sense of ethical responsibility in healthcare practices. It would be valuable to explore the specific content and teaching methods of these courses to better understand their impact on students’ ethical orientations in business. Implementing case studies or simulations that address ethical dilemmas specific to medical business situations might enhance students’ ethical awareness and critical thinking skills.

The emphasis on moral objectivism among medical students suggests a prioritization of professional integrity and patient welfare in business decisions. This alignment with universal moral standards may reflect their commitment to maintaining ethical standards, even in commercial contexts. Understanding how medical ethics principles transfer to business environments could have broader implications for enhancing ethical conduct across various sectors. 

The obtained results highlight the differences between medical and management students regarding their attitudes toward business ethics, specifically in terms of machiavellianism, ethical relativism, social darwinism, and legalism. These findings suggest that the ethics courses taught by the respective faculties contribute to the observed differences in business philosophies between medical and management students. Similarly, one should also take into consideration educational backgrounds. Medical students may receive specialized training that emphasizes patient care, empathy, and ethical considerations in their practice. In contrast, management students might focus more on strategic decision making and profit-oriented approaches. The distinct educational contexts might shape their ethical perspectives in business settings. 

Differences in attitudes toward business ethics might also be influenced by the organizational culture prevalent in medical and management settings. Medical institutions often emphasize patient-centered care and ethical conduct, while business organizations may prioritize financial performance and competition. The contrasting values of these environments might have contributed to the observed differences in ethical perspectives.

Understanding the differences in ethical attitudes between medical and management students highlights the importance of interdisciplinary collaboration in healthcare settings. Integrating medical and business perspectives could lead to more comprehensive and ethically sound decision making in healthcare administration and management.

Regarding the doctors, their preferred business philosophy is also moral objectivism, followed by legalism, then machiavellianism, ethical relativism, and social darwinism. Moral objectivism is the philosophical belief that moral principles are objective and independent of personal opinions or cultural norms. In other words, there are universal moral principles that apply to everyone, regardless of individual beliefs or cultural contexts.

In this study, we examined the relationship between variables such as age, sex, and ethics education and the attitude toward business ethics, but the scientific literature shows us that there are several categories of factors that can influence the attitude toward business ethics, divided into individual factors such as sex [47,62], religious beliefs [63] and religion practice [64], situational factors (managerial position and practical or societal experience (level of economic development, political ideology), etc.

For all group of students, the regression model was statistically significant for Machiavellianism, social Darwinism, ethical relativism, and legalism, but not for the philosophy found predominant in all the groups studied, i.e., moral objectivism. 

These results lead us to the idea that other variables that influence the attitude of the students and doctors participating in the study toward business ethics should be studied. There could be other reasons why both medical students and doctors adhere to the moral objectivism philosophy, for example, professional ethics, need for consistency, desire for impartiality, and upholding public trust. 

Medical students and doctors are trained to abide by a set of professional ethical principles, such as beneficence, nonmaleficence, justice, and respect for autonomy. These principles are based on the idea that healthcare professionals have a moral obligation to act in the best interest of their patients. Moral objectivism aligns with this professional code of ethics.

Healthcare professionals need to make ethical decisions on a daily basis, often under time constraints and with limited information. Moral objectivism provides a consistent framework for making these decisions based on universal moral principles.

Moral objectivism emphasizes impartiality and treating all individuals equally, regardless of personal biases or preferences. This is important for healthcare professionals, who must provide care to all patients without discrimination.

Healthcare professionals are expected to act in the best interest of their patients and to maintain the public trust. By adhering to a philosophy of moral objectivism, medical students and doctors can demonstrate their commitment to ethical principles and professionalism, which can enhance public trust and confidence in the healthcare system.

We also observed an underestimation of ethical relativism both in the group of medical students and in the group of doctors. Ethical relativism is the view that moral principles are subjective and relative to each individual or culture. Healthcare professionals may reject this view because it could lead to a situation where different professionals have different moral standards, leading to confusion and inconsistency in patient care. 

The legalism dimension ranked second among doctors in terms of their preference for a business philosophy and last among medical students. One possible explanation for this result could be related to changes in life experience and cognitive development. For example, doctors may have more life experience and a greater awareness of the complexities and nuances of legal systems, leading to more nuanced beliefs about the role of law in society. Additionally, age-related changes in cognitive abilities such as reasoning and judgment may also have contributed to the differences in legalism scores among age groups. However, further research would be needed to fully explore the reasons behind this finding.

In our study, the age variable proved to have no influence on the dimensions of business ethics. But, the relatively small difference between the average age of doctors (31.5 years) and the average age of medical students (24.4 years) could have been the cause of this result and can be considered a limitation of our study. 

We mention here that there are other limitations to our results: the number of respondents and the sampling technique. Participants were recruited through targeted sampling techniques, such as reaching out to students and healthcare professionals via professional networks, organizations, and universities. This method of recruiting participants by convenience sampling may introduce biases, and this aspect should be taken into account.

Ethics education/instruction was found to be an important factor influencing attitudes toward business ethics. This study argues that ethics is intrinsic to any educational action and that the values transmitted by educational institutions guide behavior. Overall, the study provides valuable insights into the attitude toward business ethics among Romanian medical students and doctors, highlighting the role of education in shaping these attitudes. 

## 5. Conclusions

In conclusion, this study investigated the influence of ethics education, sex, and age on machiavellianism (MK), social darwinism (SD), moral objectivism (MO), ethical relativism (ER), and legalism (Leg), the live dimensions that characterize business ethics. The results showed that ethics education was a significant predictor of low machiavellianism, social darwinism, and ethical relativism. Sex was found to be a significant predictor of machiavellianism and social darwinism for the entire group of medical and management students taken together. Age did not significantly predict any of the dependent variables. The regression model for moral objectivism was not significant, meaning that none of the independent variables was found to be a significant predictor of moral objectivism. 

Overall, the results of the study suggest that ethics education plays a crucial role in shaping the attitudes of medical students and doctors toward business ethics. The study underscores the importance of designing effective ethics education programs that are tailored to the needs of medical students and doctors. Such programs can help promote ethical behavior in the medical profession and contribute to the development of a more responsible and sustainable healthcare system.

## Figures and Tables

**Table 1:** Means and standard deviations of ethical philosophies.

Ethical Philosophy	M	SD	n	SE_M_	Min	Max	Skewness	Kurtosis
Machiavellianism	2.52	0.49	53	0.06	1.17	3.58	−0.12	0.001
Ethical Relativism	2.41	0.64	53	0.09	1	3.67	−0.25	−0.45
Social Darwinism	2.34	0.47	53	0.06	1	3.38	−0.44	0.33
Moral Objectivism	3.21	0.63	53	0.09	1	4.43	−0.79	1.44
Legalism	2.34	1.02	53	0.07	1	5	0.39	−0.49

**Table 2:** Mean values and standard deviations for business philosophies for the doctor group.

Ethical Philosophy	M	SD	n	SE_M_	Min	Max	Skewness	Kurtosis
Machiavellianism	2.6	0.48	192	0.03	1	4.33	0.53	1.44
Ethical Relativism	2.5	0.55	192	0.04	1	3.67	−0.15	−0.57
Social Darwinism	2.3	0.47	192	0.03	1	3.88	0.1	0.51
Moral Objectivism	3.25	0.52	192	0.04	1	4.57	−0.37	1.54
Legalism	2.77	1.2	192	0.09	1	5	0.19	−1

## Data Availability

Data available on demand.

## Appendix A

**Table A1:** Mean (M) and standard deviation (SD) of ethical philosophies and the results of ANOVA.

Philosophy	Med. Stud.		Mg. Stud.		F (Snedecor)
	M	SD	M	SD	
Machiavellianism	2.52	0.49	3.09	0.43	*F*(1, 159) = 56.69, *p* < 0.001
Ethical Relativism	2.41	0.64	2.83	0.56	*F*(1, 159) = 19.90, *p* < 0.001
Moral Objectivism	3.21	0.63	3.18	0.48	*F*(1, 159) = 0.42, *p* = 0.519
Social Darwinism	2.34	0.47	2.6	0.43	*F*(1, 159) = 10.39, *p* = 0.002
Legalism	2.34	1.02	2.88	1.01	*F*(1, 159) = 9.12, *p* = 0.003
